# Epidemiologic evaluation of calcium oxalate urolithiasis in dogs in the United States: 2010‐2015

**DOI:** 10.1111/jvim.15613

**Published:** 2019-08-30

**Authors:** Vachira Hunprasit, Pamela J. Schreiner, Jeffrey B. Bender, Jody P. Lulich

**Affiliations:** ^1^ Faculty of Veterinary Science Chulalongkorn University Bangkok Thailand; ^2^ Division of Epidemiology and Community Health School of Public Health, University of Minnesota Minneapolis Minnesota; ^3^ Division of Environmental Health Sciences School of Public Health, University of Minnesota Minneapolis Minnesota; ^4^ Veterinary Clinical Sciences College of Veterinary Medicine Minneapolis Minnesota

**Keywords:** calculus, dog, epidemiology, North America, risk factor

## Abstract

**Background:**

Positive health implications of early recognition of calcium oxalate (CaOx) urolithiasis include increased opportunity for nonsurgical removal, early dietary modification to minimize urolith growth, early removal to avoid urinary obstruction, and early recognition of genetic and metabolic diseases before they contribute to additional morbidity.

**Objectives:**

To identify high‐ and low‐risk dog breeds for CaOx uroliths and to determine the relationship of age and sex to the development of CaOx uroliths.

**Animals:**

Calcium oxalate urolith submissions between 2010 and 2015.

**Methods:**

A comparative cross‐sectional study was conducted to identify high‐ and low‐risk breeds for CaOx uroliths by comparing cases to multiple comparison groups. At‐risk breeds were identified if odds ratios were significant (*P* value <.05) across all comparison groups.

**Results:**

Of 258 898 urolith submissions, 124 285 were CaOx. Calcium oxalate was identified in 212 breeds. Twelve breeds were identified as high‐risk breeds, and 14 breeds were identified as low‐risk breeds. All high‐risk breeds were small dog breeds, and all low‐risk breeds were medium to large dog breeds. Overall, the mean age ± standard deviation of the first CaOx urolith was 8.4 ± 2.8 years.

**Conclusions and Clinical Importance:**

To achieve the health benefits of preclinical evaluation, breeds at high risk for CaOx urolithiasis should be screened at 5 to 6 years of age, which is 2 to 3 years before likely development of clinical urolithiasis.

**Abbreviations**
CaOx: calcium oxalate
OR: odds ratio

## 1 INTRODUCTION

Calcium oxalate (CaOx) is the most frequent urolith submitted for analysis from dogs in the United States.^1^, ^2^ The positive health implications of early recognition of CaOx urolithiasis include increased opportunity for nonsurgical removal, early dietary modification to minimize urolith growth, early removal to avoid urinary obstruction, and early recognition of potential genetic and metabolic diseases such as hypercalcemia before these diseases contribute to additional morbidity. To achieve these health benefits, CaOx urolithiasis will need to be recognized early, before development of clinical disease. One method of recognizing which dogs will benefit most from preclinical evaluation is an understanding of which dogs are at greatest risk for CaOx urolithiasis and when in a dog's lifetime risk is highest.

The optimal comparison group in urolithiasis research to determine dog breeds at risk for urolith formation has not been established. In 2 case‐control studies, the comparison groups were hospital‐based nonurinary tract disease dogs.^3^, ^4^ In another study, the comparison group was dogs with struvite uroliths.^5^ Although the studies reported breeds from which CaOx uroliths were most frequently submitted for analysis, the majority of these studies did not account for breed popularity and hence the size of the population at risk. The objective of our comparative cross‐sectional study was to identify high‐ and low‐risk breeds for CaOx urolith occurrence and to determine the relationship of age and sex to the development of CaOx uroliths by comparing cases to multiple comparison control groups.

## 2 MATERIALS AND METHODS

### 2.1 Calcium oxalate case population

Urolith submissions to the Minnesota Urolith Center, University of Minnesota, between January 1, 2010, and December 31, 2015, were reviewed. Records were eligible for inclusion if dogs resided in the United States, and uroliths were ≥70% CaOx as determined by quantitative analysis.^6^ Records of dogs with compound uroliths having a central core that was ≥70% CaOx also were eligible for inclusion.

### 2.2 Comparison populations

Three canine populations were used for comparison: (1) non‐CaOx urolith formers uroliths of which were analyzed by the mineral analysis laboratory during the same period as the CaOx cases, (2) hospital population of dogs without prior or current urinary disease that visited the Veterinary Medical Center, University of Minnesota during the same period as the CaOx cases, and (3) dogs in a breed popularity survey performed by the Dog Breed Info Center between September 7, 2013, to January 12, 2016 (Dog Breed Info Service: http://www.dogbreedinfo.com/articles/populardogbreeds2016.htm).

### 2.3 Dog‐level variables

From the urolith populations (CaOx cases and non‐CaOx dogs), breed (as categorized by the American Kennel Club, www.akc.org/dog-breeds), sex (male or female) and age at urolith removal were obtained from urolith submission records. From the hospital population, breed, sex (male or female), and age at first visit were extracted from the medical records. Only information regarding the breed was available from the survey population.

### 2.4 Statistical analyses

Means and standard deviation (SD) were determined for age at first urolith occurrence or first hospital visit; differences in mean age between the CaOx group and the non‐CaOx group, and between the CaOx group and the hospital group, were compared by Student's *t* test for 2 independent samples after ascertaining that age was normally distributed by Q‐Q plot and skewness. Age groups were further categorized into quartiles based on the frequency distribution among CaOx cases: <6, 6‐8, 8‐10, and >10 years.

Logistic regression was used to estimate odds ratios (OR) and 95% confidence intervals (CI) for breed using the CaOx urolith group for cases and the non‐CaOx urolith group, hospital admission group, or breed survey as control groups. Odds ratios for age categories and sex used the non‐CaOx urolith and hospital admissions data as the control group because age and sex were not available for the breed survey. When breed was the exposure of interest, each breed was considered with all other breeds (including mixed breed) serving as the reference group for that analysis. High‐risk breeds were identified if ORs from all 3 comparison groups were >1.00 and statistically significant based on a 2‐tailed *P* value <.05. Likewise, low‐risk breeds were identified if ORs from all 3 comparison groups were <1.00 and statistically significant. Analyses only included dogs with data for breed, age, and sex, and not dogs with incomplete data. Statistical analysis was performed using SAS/STAT software (SAS Institute Inc, Cary, North Carolina).

## 3 RESULTS

Between January 1, 2010, and December 31, 2015, 258 898 urolith submissions from dogs were analyzed by the mineral analysis laboratory. Forty‐eight percent (124 285) of urolith submissions were classified as CaOx (365 upper urinary tract uroliths [ie, kidney and ureteral uroliths] and 123 920 lower urinary tract uroliths [ie, bladder and urethral uroliths]). The number of non‐CaOx urolith submissions during the same period was 134 613 (98 upper urinary tract uroliths and 134 515 lower tract uroliths). The hospital comparison population consisted of 35 658 dogs without urinary tract disease during the same period. The breed popularity study provided information from 12 003 dogs.

### 3.1 Breed

Calcium oxalate uroliths were identified in 212 breeds, including dogs of mixed breed. Twelve dog breeds were classified as high‐risk breeds for developing CaOx uroliths. All high‐risk breeds were small breed dogs. However, the magnitude of the association differed depending on the comparison population (Table 1).

**Table 1:** Odds ratios (OR) and 95% confidence intervals (CI) for dog breeds at high and low risk of forming calcium oxalate (CaOx) uroliths (n = 124 285) compared to three groups: non‐CaOx uroliths (n = 134 613), hospital admissions (n = 35 658), and a breed survey (n = 12 003)

Breed groups	CaOx versus non‐CaOx urolith forming dogs	CaOx versus hospital admissions	CaOx versus breed survey
	OR (95% CI)	OR (95% CI)	OR (95% CI)
High‐risk breeds^a^			
Bichon Frise	1.06 (1.03‐1.10)	4.82 (4.42‐5.27)	12.03 (10.24‐16.26)
Brussels Griffon	3.10 (2.61‐3.70)	3.82 (2.73‐5.34)	3.67 (2.11‐6.37)
Cairn Terrier	3.24 (2.89‐3.65)	2.16 (1.82‐2.56)	2.90 (2.10‐4.02)
Chihuahua	2.19 (2.15‐2.36)	2.70 (2.49‐2.94)	1.15 (1.05‐1.27)
Jack Russell	2.06 (1.96‐2.22)	2.01 (1.80‐2.25)	2.01 (1.71‐2.40)
Japanese Chin	1.34 (1.16‐1.54)	3.46 (2.46‐4.87)	3.02 (1.77‐5.15)
Lhasa Apso	2.65 (2.54‐2.87)	5.29 (4.60‐6.10)	8.83 (6.69‐12.31)
Maltese	2.80 (2.71‐3.03)	3.29 (2.96‐3.65)	5.34 (4.42‐6.85)
MN Pinscher	3.41 (3.07‐3.84)	1.62 (1.40‐1.86)	1.58 (1.25‐1.99)
MN Schnauzer	2.03 (1.97‐2.08)	8.20 (7.56‐8.89)	12.00 (11.76‐16.36)
Pomeranian	3.31 (3.25‐3.61)	4.56 (4.10‐5.08)	4.27 (3.60‐5.07)
Yorkshire Terrier	2.06 (2.09‐2.23)	3.60 (3.36‐3.86)	4.46 (4.23‐5.46)
Low‐risk breeds^a^			
American Bulldog	0.10 (0.07‐0.14)	0.06 (0.04‐0.09)	0.03 (0.02‐0.04)
American Staffordshire	0.37 (0.27‐0.49)	0.04 (0.03‐0.06)	0.06 (0.04‐0.08)
Australian Cattle Dog	0.24 (0.18‐0.31)	0.15 (0.11‐0.21)	0.06 (0.04‐0.08)
Australian Shepherd	0.48 (0.41‐0.54)	0.20 (0.17‐0.23)	0.22 (0.17‐0.26)
Basset Hound	0.46 (0.40‐0.52)	0.32 (0.27‐0.38)	0.53 (0.39‐0.69)
Beagle	0.47 (0.43‐0.51)	0.32 (0.29‐0.55)	0.41 (0.34‐0.47)
Border Collie	0.25 (0.20‐0.30)	0.05 (0.04‐0.07)	0.32 (0.21‐0.47)
Boxer	0.27 (0.23‐0.32)	0.06 (0.05‐0.07)	0.06 (0.05‐0.06)
Chow Chow	0.38 (0.30‐0.48)	0.30 (0.22‐0.41)	0.21 (0.14‐0.29)
French Bulldog	0.42 (0.35‐0.49)	0.19 (0.16‐0.23)	0.32 (0.23‐0.40)
German Shepherd	0.27 (0.22‐0.32)	0.02 (0.01‐0.02)	0.028 (0.023‐0.034)
Golden Retriever	0.22 (0.18‐0.25)	0.02 (0.02‐0.02)	0.08 (0.06‐0.09)
Labrador Retriever	0.14 (0.12‐0.15)	0.01 (0.01‐0.02)	0.067 (0.059‐0.076)
Siberian Husky	0.55 (0.46‐0.65)	0.16 (0.13‐0.19)	0.12 (0.09‐0.15)

a Only breeds in which the direction of association was statistically significant (*P* value <.05) in all three comparisons were reported.

Fourteen dog breeds were considered as low‐risk breeds for CaOx uroliths. Most low‐risk breeds were medium and large breeds except for 2 small dog breeds, Beagle and French Bulldog (Table 1).

### 3.2 Sex

Calcium oxalate uroliths were submitted more often from male dogs than from female dogs (73.1% versus 26.9%) and were more common in neutered (85.5%) than intact dogs (14.5%). Comparing CaOx urolith dogs to the non‐CaOx urolith dogs, males had 12 times greater odds of developing CaOx uroliths than did female dogs (Table 2). Comparing CaOx urolith dogs to the hospital population without urinary tract disease, males were at only 2.5 times greater odds of developing CaOx uroliths than were females (Table 2).

**Table 2:** Number (n), odds ratio (OR), and 95% confidence interval (CI) for age and sex of dogs with calcium oxalate (CaOx) uroliths compared to two comparison groups: non‐CaOx uroliths and hospital admissions

Variables	CaOx (n)	Non‐CaOx (n)	OR (95% CI) for CaOx versus non‐CaOx	Hospital admission (n)	OR (95% CI) for CaOx versus hospital admission
Age, y					
<6	23 136	65 850	1.00 (Ref)	15 759	1.00 (Ref)
6–8	28 878	29 585	2.77 (2.71‐2.84)	5434	3.62 (3.49‐3.75)
8‐10	32 043	19 717	4.62 (4.51‐4.73)	5516	3.95 (3.82‐4.09)
>10	36 147	14 285	7.20 (7.02‐7.38)	8444	2.91 (2.82‐3.00)
Sex					
Female	33 025	108 398	1.00 (Ref)	17 073	1.00 (Ref)
Male	89 726	24 437	12.05 (11.82‐12.27)	18 312	2.53 (2.47‐2.59)

Abbreviation: Ref, reference population.

### 3.3 Age

The mean age of dogs with CaOx uroliths was 8.6 ± 2.7 years, which was significantly older than non‐CaOx urolith dogs (6.3 ± 2.9 years, *P* value <.001) and hospital population dogs (6.9 ± 4.3 years, *P* value <.001). Male dogs with CaOx uroliths were significantly younger than female dogs with CaOx uroliths (8.5 ± 2.8 years versus 8.8 ± 2.6 years, *P* value <.001). When comparing CaOx urolith dogs with non‐CaOx urolith dogs, the odds of developing CaOx uroliths were positively associated with higher ages (Table 2). Dogs >10 years of age had the highest odds (OR, 7.20; 95% CI, 7.02‐7.38) for developing CaOx uroliths compared to dogs <6 years of age. Similarly, when comparing CaOx urolith dogs with hospital dogs, the odds of developing CaOx uroliths were significantly higher relative to dogs <6 years of age for all age quartiles. The odds of developing CaOx uroliths were highest for the age group 8‐10 years (OR, 3.95; 95% CI, 3.82‐4.09), and lowest for age group >10 years of age (OR, 2.91; 95% CI, 2.82‐3.00; Table 2).

First‐time CaOx uroliths were reported for 70.3% (87 332) of dogs. The mean age of first‐time formers was 8.4 ± 2.8 years, which was 10 months younger than dogs with a history of previous uroliths (9.2 ± 2.5 years). First‐time male CaOx formers were younger than first‐time female CaOx formers (8.3 ± 2.8 versus 8.6 ± 2.6 years; *P* value <.001). Mean ages of first‐time CaOx urolith formers in high‐risk breeds are presented by breed and sex in Table 3.

**Table 3:** Mean *±* SD age (year) of high‐risk breeds at first occurrence of calcium oxalate uroliths in males and females

Breed	First episode of CaOx urolith			
	Male		Female	
	n	Mean age (y)	n	Mean age (y)
Bichon Frise	3852	8.9 ± 2.6	1451	9.0 ± 2.5
Brussels Griffon	295	7.3 ± 2.6	32	6.9 ± 2.3
Cairn Terrier	473	8.5 ± 2.7	307	8.8 ± 2.4
Chihuahua	3263	8.0 ± 2.7	969	8.4 ± 2.6
Jack Russell Terrier	1606	9.2 ± 2.7	388	9.3 ± 2.6
Japanese Chin	251	7.7 ± 2.3	62	7.9 ± 2.6
Lhasa Apso	1848	8.8 ± 2.8	663	9.2 ± 2.7
Maltese	2042	8.4 ± 2.7	1090	8.3 ± 2.5
Miniature Pinscher	801	7.9 ± 2.6	89	8.7 ± 2.3
Miniature Schnauzer	6322	8.4 ± 2.7	4047	8.3 ± 2.6
Pomeranian	3004	7.5 ± 2.6	869	8.1 ± 2.6
Yorkshire Terrier	6318	7.6 ± 2.7	1041	7.9 ± 2.4

## 4 DISCUSSION

Identifying breeds at high‐ and low‐risk for CaOx uroliths is important for clinicians to determine which breeds should be screened for disease. In our study, 12 breeds were identified to be at high‐risk (Table 1). Fourteen breeds were identified to be low‐risk breeds for CaOx urolith formation. These results are similar to those of other studies of dogs residing in the United States.^1^, ^3^ However, using different comparison populations provided additional insight. Bichon Frise, Miniature Schnauzer, Lhasa Apso, Pomeranian, Cairn Terrier, Yorkshire Terrier, and Maltese were identified in prior studies. Two additional breeds identified in our study, the Jack Russell Terrier and Chihuahua, also were identified in another study.^3^ However, the Japanese Chin, Miniature Pinscher, and Brussels Griffon were not identified previously. The Shih Tzu and Keeshond were identified in previous studies but were not significant across all 3 of our comparison groups.^1^ However, these breeds can be included in the list of dogs to monitor for CaOx uroliths if other risk factors such as a family history of CaOx uroliths or persistent CaOx crystalluria are identified. A study of dogs with CaOx uroliths residing in the United Kingdom identified similar high‐risk breeds, indicating that our results may be applicable to other geographic areas outside the United States.^7^ It was not surprising that the high‐risk breeds identified in our study also were the highly prevalent breeds reported in several other studies.^8^, ^9^, ^10^, ^11^, ^12^

All high‐risk breeds were small‐breed dogs. Our finding was similar to other studies in which small‐breed dogs were at highest risk for CaOx urolith development.^4^, ^5^ This observation can be partly explained by the possibility that small‐breed dogs tend to be hypercalciuric compared to large‐breed dogs.^13^ Hypercalciuria is a pathophysiological risk factor for CaOx precipitation because increased calcium excretion increases urinary CaOx saturation.

Calcium oxalate uroliths were submitted significantly more often from males than from females. Our observation is in agreement with results of previous studies.^1^, ^3^, ^4^, ^7^, ^8^, ^9^, ^10^, ^12^, ^13^, ^14^, ^15^, ^16^ The reason male dogs were predisposed for CaOx uroliths is not well understood. Results from studies in other species suggest several possibilities. In humans, men have a higher incidence of CaOx urolithiasis because men generally excrete more calcium, oxalate, and uric acid than do women.^17^ Women are postulated to have a lower incidence of CaOx urolith development because of an estrogen‐dependent increase in urine citrate concentration, a chelator of calcium, and a decrease in urine calcium concentration.^18^ The high percentage of neutered CaOx dogs in our study may indicate that CaOx formation is less associated with hormonal differences between the sexes of dogs and opens the possibility of genetically acquired traits on sex‐differentiating chromosomes. Lastly, females may be underrepresented because a shorter and wider urethra may allow uroliths to pass more easily and evade detection.

The risk of CaOx urolith formation was higher with advancing age. Compared to non‐CaOx urolith‐forming dogs, the odds were higher with each higher age quartile. This result was likely biased because of the high number of struvite urolith‐forming dogs in this comparison group. Struvite uroliths often form in younger dogs more than in older dogs, which magnifies the effect for older CaOx dogs.^19^, ^20^ Using the hospital comparison group, the risk for CaOx was highest for dogs that were 8‐10 years of age; for older dogs, the risk was lower. This observation has been reported in other studies that considered age as continuous^4^ and categorical variables.^3^ This finding may imply that the risk for CaOx uroliths decreases in dogs >10 years of age. This apparent decrease also may be due to unmeasured factors that confound the association such as diet, medications, and comorbidities.

Knowing the age when dogs develop their first CaOx urolith can be used to determine when to screen high‐risk dogs for CaOx uroliths. In our study, the mean age of first‐time urolith formers was 8.4 ± 2.8 years. Results from a multihospital study determined that the mean age of first‐time CaOx urolith formers was 7.5 ± 3.0 years.^4^ This difference may be attributable to the different definition of age between the 2 studies. In the multihospital study, age was identified as age at urolith detection. In our study, age at urolith removal was used. If we assume that all uroliths were submitted to the mineral analysis laboratory, the difference of 10 months likely accounted for dietary and medical treatment to manage uroliths before removal.

The positive health implications of early recognition of CaOx urolithiasis includes early intervention to minimize disease morbidity. In addition, CaOx uroliths detected early often are small. Small uroliths are removed more easily by nonsurgical methods. In 1 study, the optimal urolith size for removal by voiding urohydropropulsion was <3 mm in small male dogs.^21^, ^22^ Therefore, diagnosing CaOx uroliths before clinical disease should decrease the expense of urolith removal as well as the complications of surgery to remove uroliths.

To detect CaOx uroliths before clinical signs occur, we recommend annual medical imaging starting at 5 to 6 years of age for breeds at high risk for CaOx uroliths. One SD below the mean age of first‐time urolith formers was 5.6 years. Using this age, 84% of dogs would have been screened and diagnosed with urolithiasis before urolith removal, but 16% of urolith formers already may have developed clinical disease, necessitating urolith removal. Initiating screening at 2.8 years of age, which is 2 SDs below the mean age of first‐time CaOx urolith formers, would screen and diagnose 97.5% of dogs before urolith removal. However, 2.5% of stone formers already may have developed clinical disease necessitating urolith removal. Calcium oxalate urolithiasis is rarely a life‐threatening disease. Therefore, to minimize evaluating dogs too early, we suggest starting medical screening of high‐risk dogs at 5.6 years of age. However, there is minimal risk in evaluating dogs sooner. Initiating evaluation at younger ages should be considered for dogs with additional risk factors (eg, family history of CaOx urolithiasis, persistent CaOx crystalluria). Likewise, the mean ages of several high‐risk breeds (Brussels Griffons, Pomeranians, and Yorkshire Terriers) were approximately a year earlier than the mean age of all high‐risk breeds (Table 3). It is logical to assume that these breeds also should be screened at an earlier age.

In 1 study, the median age of CaOx urolith recurrence in miniature Schnauzers was 1.8 years.^23^ We interpret these findings to indicate that once enough risk factors are present, it takes approximately 1.8 years before patients develop clinical disease. Therefore, we recommend annual screening to encompass a shorter interval to detect dogs before clinical disease when uroliths are smaller.

Several imaging modalities are available for screening dogs for CaOx uroliths. We prefer survey radiography because of its ability to evaluate all portions of the urinary tract for this radiopaque urolith.^24^ Although ultrasonography is more sensitive at detecting urocystoliths, ultrasonography is limited in its ability to detect urethroliths in the distal urethra.^25^ Similarly, ultrasonography is inferior at assisting prediction of urolith composition, which is a function of the radiographic density of uroliths.

Our study had several limitations. Because of its retrospective nature, not all epidemiologic data were available for each urolith submission. Some owners and veterinarians may have misclassified known breeds as mixed breed, and the reverse. Similar errors may have occurred when entering sex and age. However, because of the large sample size and the randomness of errors, we hypothesize that misclassifications would not have affected our study's conclusions. Uroliths naturally voided by the dog, medically dissolved by the veterinarian, or not submitted for analysis would not have been included in the analysis. Almost all the CaOx submissions were from uroliths removed from the lower urinary tract. Therefore, the study results would be more reliable for the development of lower urinary tract CaOx uroliths than for the development of upper urinary tract CaOx uroliths.

In our study, the Bichon Frise, Brussels Griffon, Cairn Terrier, Chihuahua, Jack Russell, Japanese Chin, Lhasa Apso, Maltese, Miniature Pinscher, Miniature Schnauzer, Pomeranian, and Yorkshire Terrier were identified as high‐risk breeds for CaOx uroliths. Although the age of first‐time male CaOx urolith formers was significantly lower than that of the first‐time female CaOx urolith formers, this difference was clinically similar. Based on our findings, we recommend that high‐risk breeds of either sex begin urolith screening at 5 to 6 years of age to detect uroliths before development of clinical signs and urethral obstruction and at a time in urolith development when nonsurgical removal by voiding urohydropropulsion and basket retrieval is feasible.

## CONFLICT OF INTEREST DECLARATION

Authors declare no conflict of interest.

## OFF‐LABEL ANTIMICROBIAL DECLARATION

Authors declare no off‐label use of antimicrobials.

## INSTITUTIONAL ANIMAL CARE AND USE COMMITTEE (IACUC) OR OTHER APPROVAL DECLARATION

Authors declare no IACUC or other approval was needed.

## HUMAN ETHICS APPROVAL DECLARATION

Authors declare human ethics approval was not needed for this study.
